# Phosphatidylethanol for Monitoring Alcohol Use in Liver Transplant Candidates: An Observational Study

**DOI:** 10.3390/jcm9093060

**Published:** 2020-09-22

**Authors:** Pablo Barrio, Antoni Gual, Anna Lligoña, Lidia Teixidor, Wolfgang Weinmann, Michel Yegles, Friedrich M. Wurst

**Affiliations:** 1 Grup Recerca Addicions Clínic (GRAC-GRE), Department of Psychiatry, Clinical Institute of Neuroscience, Hospital Clínic i Universitari de Barcelona, Universitat de Barcelona, IDIBAPS, RTA (RETICS), Villarroel, 170, 08036 Barcelona, Spain; tgual@clinic.cat (A.G.); alligona@clinic.cat (A.L.); lteixido@clinic.cat (L.T.); 2 Institute of Forensic Medicine, University of Bern, 3012 Bern, Switzerland; Wolfgang.Weinmann@irm.unibe.ch; 3 Laboratoire National de Santé, Service de Toxicologie, 3555 Dudelange, Luxembourg; michel.yegles@lns.etat.lu; 4 Psychiatric University Hospital, 4002 Basel, Switzerland; frieder.wurst@gmx.de

**Keywords:** liver transplatation, phosphatidylethanol, ethylglucuronide, alcohol dependence

## Abstract

Liver transplantation remains an essential procedure for many patients suffering from alcoholic liver disease. Alcohol use monitoring remains paramount all through the stages of this complex process. Direct alcohol biomarkers, with improved specificity and sensibility, should replace traditional indirect markers. Phosphatidylethanol (PEth) has been recently tested in alcoholic liver disease patients, but more evidence is needed, especially in comparison with other direct biomarkers. We conducted an observational study among patients awaiting liver transplantation. We analyzed Peth in blood, ethylglucuronide (EtG) in hair and urine and ethylsulphate (EtS) in urine, using mass spectrometry methods. In addition, transaminases, and self-reports were analyzed. A total of 50 patients were included (84% men, mean age 59 years (SD = 6)). 18 patients (36%) screened positive for any marker. Self-reports were positive in 3 patients. EtS was the biomarker with more positive screens. It also was the most frequently exclusive biomarker, screening positive in 7 patients who were negative for all other biomarkers. PEth was positive in 5 patients, being the only positive biomarker in 2 patients. It showed a false negative in a patient admitting alcohol use the previous week and screening positive for EtG and EtS. Hair EtG was positive in 3 patients who had negative Peth, EtG. EtG did not provide any exclusive positive result.A combination of biomarkers seems to be the best option to fully ascertain abstinence in this population. Our study suggest EtS might also play a significant role.

## 1. Introduction

Alcoholic liver disease (ALD) remains the most frequent etiology in life-threatening liver disease in a majority of European countries and the US [1,2]. Despite some controversies [3,4], especially in acute hepatitis unresponsive to medical treatment [5], liver transplantation (LTX) is a well established and valuable procedure in ALD, especially in end-stage cirrhosis [6,7].

One of the critical issues surrounding LT in alcohol patients is abstinence monitorization, since abstinence seems to be the gold standard when it comes to treatment aims. That partly explains the 6-month abstinence rule frequently required prior to LT. Again, the rule does not come without controversy [8,9,10], with some authors arguing in favor of a more dimensional evaluation of the relapse risk, instead of a binary and time-constrained assessment [11].

Therefore, it seems almost self-evident that in a majority of ALD patients undergoing or waiting for LT, abstinence assessment and monitorization remains critical, and also challenging. Traditional indirect alcohol biomarkers remain far from accurate [12,13,14]. Moreover, they are easily affected by other medical conditions and variables such as age and sex. On the other hand, self-reports frequently tend to underreport drinking [15,16,17,18].

In this regard, direct alcohol biomarkers (i.e., biomarkers formed in the presence of ethanol) have consistently demonstrated a better diagnostic validity, both in terms of sensitivity and specificity, for the monitorization of abstinence [19,20].

The two most validated biomarkers in ALD patients have been ethylglucuronide (EtG) and phosphatydilethanol (PEth). Two studies conducted with EtG clearly showed its better accuracy compared to traditional biomarkers [16,17]. Also, when compared to self-reports, EtG disclosed a higher rate of drinking. Similarly, PEth has been tested in two studies with ALD patients [21,22], and two studies including both pre- and post-transplant patients [23,24]. Globally, they all seem to suggest that PEth provides significant added value to monitorization procedures in ALD patients, since, similarly to other direct alcohol biomarkers, PEth displays a higher sensitivity when compared to self-reports or traditional, non-direct biomarkers. Also, it can be obtained directly from blood, which makes it much less prone to contamination or manipulation. However, more studies with more patients are needed to confirm these preliminary results. Also, more direct comparisons between direct alcohol biomarkers in these populations are also warranted to better evaluate the diagnostic performance of the different direct alcohol biomarkers. Here, we present the results of an observational study conducted among liver transplant candidates undergoing psychiatric evaluation and abstinence monitoring. The main objective of the study was to evaluate the frequency of a positive PEth result in this population, as well as comparing PEth performance to that of other biomarkers, especially EtS, EtG in urine and hair.

## 2. Methods

### 2.1. Study Design

This was a single-center, cross-sectional study conducted in a big urban tertiary hospital in Barcelona, Spain, between November 2017 and January 2019. Results were completely anonymous and patients were reassured about the information being confidential, so their responses are not being shared with any clinical members of the research team, nor with the transplant program personnel. Ethics approval was obtained from the Clinic Hospital Ethics Committee. All participants signed informed written consents before study enrollment.

### 2.2. Study Population and Procedure

Patients with alcoholic liver disease must attend a psychiatric consultation before being enrolled in the transplantation list, usually conducted in an outpatient facility. The attending psychiatrist was responsible for consecutively offering study participation to eligible patients.

In order to be eligible, patients had to be diagnosed with an alcohol use disorder and alcoholic liver disease, and be willing to sign informed written consent. Upon acceptance, patients completed the Alcohol Use Disorder Identification Test (AUDIT) [25] and the Timeline Follow Back (TLFB) [26] for the preceding 28 days. The AUDIT is a 10-item screening tool widely used to assess drinking amount, frequency and consequences. For screening purposes, a cut-off of 8 is normally established between low and medium risk. The TLFB is a calendar method used to retrospectively record all the alcohol units ingested in the preceding 28 days. After that, blood and urine samples were obtained. If possible, hair samples were also extracted.

### 2.3. Biomarker Assessment

PEth 16:0/18:1 and PEth 16:0/18:2 were analyzed in Dried Blood Spots (DBS) from venous blood (20 μL). D5-PEth 16:0/18:1 and D5-PEth 16:0/18:2 were used as internal standards. DBS were stored at −20 °C (range of storage time from 7 to 90 days).

Deuterated standards were synthesized in our laboratory from PC 16:0/18:1 and PC 16:0/18:2 and D6-ethanol catalyzed by phospholipase D (Schröck et al., 2016a). For DBS 15 preparation, 20 μL of whole blood were pipetted on filter cards (GR2261004, PKI 226 Bioanalysis Card, Perkin Elmer, Rodgau, Germany) and were dried for a minimum of 3 h prior to extraction. PEth was extracted from DBS with 500 μL of methanol (10 min). The supernatant was transferred to a vial and evaporated to dryness under a stream of nitrogen at 50 °C. The residue was redissolved in 200 μL of mobile phase A (ammonium acetate (10 mM)/acetonitrile (30:70, *v*/*v*)). An aliquot of 80 μL was injected into the online-SPE-LC-MS/MS system. A previously published validated method for PEth analysis in whole blood samples was modified for DBS by use of a calibration range of 20–2000 ng/mL (Schröck et al., 2016b). Limits of quantitation (LoQ) for PEth 16:0/18:1 and PEth 16:0/18:2 were 20 ng/mL. The analysis was performed with a QTrap 3200 tandem mass spectrometer with a turbo ionspray source (Sciex, Toronto, Canada). After trapping with a Synergi Polar-RP column (20 × 2 mm, 5 μm) the two homologues were separated with a Luna RP-C5 column (50 mm × 2 mm, 5 μm) (Phenomenex, Brechbühler, Schlieren, Switzerland) by gradient elution. Similarly, EtG and EtS were analyzed with online-SPE-LC-MS/MS system, after being obtained from urine samples.

The method used for extraction of the hair samples has been described previously (Kerekes 2013). After cleaning hair with water and acetone, pulverization and incubation for 2 h in an ultrasonic bath, a solid phase extraction was performed with OASIS Max Columns (Waters). After derivatization with heptafluorobutyric anhydride analysis was performed by GC-MS/MS in negative chemical ionization mode. The monitored ion transitions were m/z 596/213 (quantifier) and 397/213 (qualifier) for EtG, and m/z 601/213 for EtG-D5. Limit of detection (LOD) was 0.05 pg/mg hair and a lower limit of quantification (LLOQ) 0.2 pg/mg hair (Capelle 2015). Gamma-glutamyltransferase (γ-GT), alanine aminotransferase (ALT) and aspartate aminotransferase were also analyzed.

### 2.4. Statistical Analysis

Data were analyzed using SPSS (Version 23.0, SPSS Inc., Chicago, IL, USA). All metric parameters are expressed as total numbers, % or mean ± standard deviation.

## 3. Results

A total of 50 patients were consecutively included between December 2017 and October 2018. The mean age of participants was 59 years (SD = 6). A majority (84%) were men. A total of 13 participants had HCV (26%), and one participant had chronic HBV infection. HIV was present in two patients, and a lifetime history of drug use was recorded in 7 patients (14%).

Regarding alcohol screening results, 32 (64%) subjects were negative for all biomarkers. That left a total of 18 (36%) patients screening positive for any of the available markers. Table 1 shows differences for the main assessments between AUDIT positive and negative patients. Table 2 shows the main features of the study biomarkers.

**Figure 1:**
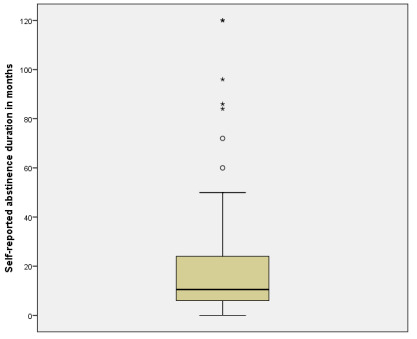
Box-plot showing self-reported abstinence duration (in months).

**Figure 2:**
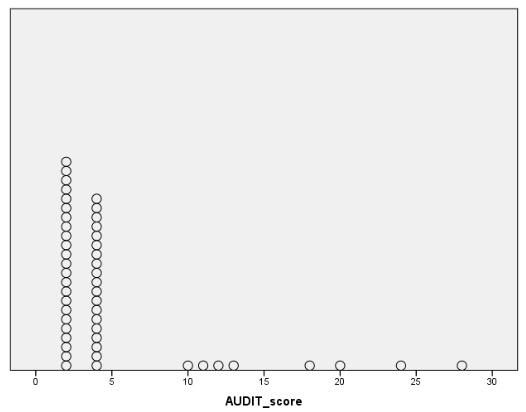
Dotplot showing Alcohol Use Disorder Identification Test (AUDIT) scores.

**Figure 3:**
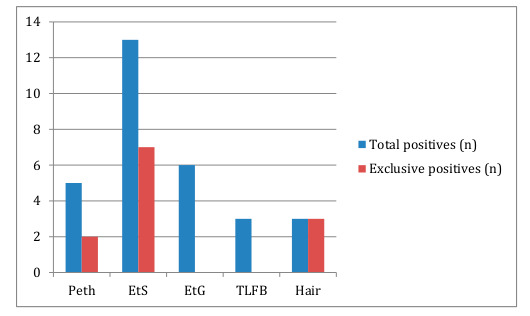
Barchart showing total and exclusive positives for each biomarker. EtG: ethylglucuronide; EtS: ethylsulfate; PeTH: phosphatydilethanol, HEtG: hair ethylglucuronide; TLFB: timeline follow back.

A total of 3 patients self-reported alcohol consumption in the previous 28 days. Equally, only these three patients reported alcohol use during the previous week. Figure 1 shows a box-plot depicting self-reported duration of abstinence in months. Figure 2 shows a dotplot depicting AUDIT scores. Figure 3 shows the total number of positives, as well as the number of exclusive positives for each biomarker.

EtS was the biomarker with more positive screens. It also was the most frequently exclusive biomarker, screening positive in 7 patients who were negative for all other biomarkers. Specifically about PEth, it was positive in 5 cases. It showed a false negative in a patient admitting alcohol use the previous week and screening positive for EtG and EtS.

Hair EtG was positive in 3 patients who had negative Peth, Etg and EtS (although one of them could not provide urine due to renal failure). TLFB and EtG did not provide an exclusive positive result.

## 4. Discussion

Assessment of the drinking status is of paramount importance all through the transplantation process. Although protocols may vary between regions and individual differences may preclude fully accurate predictions, it seems reasonable to believe that abstinence before transplantation improves the probabilities of post-transplant abstinence and compliance [31].

On the other hand, return to drinking after transplantation has been clearly linked to transplantation failure, allograft fibrosis and finally allograft loss [32], Therefore, the use of highly sensitive and specific alcohol direct biomarkers seems warranted in this population.

In our study, a selected population awaiting liver transplantation with relatively stable levels of transaminases and ggt, we found that 36% of the patients screened positive for at least one of the available biomarkers. Our major findings were: The most frequently positive biomarker was EtS, followed by EtG and PEth. Peth was positive in 5 patients, showing a false negative result in a patient admitting drinking the previous week and screening positive for EtG and EtS. These results diverge somehow from previous studies in the same population ([23], where PEth was the most frequent positive biomarker. Unfortunately, the study by Fleming and colleagues [24] did not analyze any other biomarker besides PEth and therefore no comparisons are possible. That being said, they find a 20% of positive PEth samples, which doubles our 10%, although relevant differences in sample size should be noted. Interestingly, the rates of positive self-report are similar (8 vs. 6%). Something to consider is the fact that we used 20 ng/mL as the cut-off or limit of quantification for PEth. Maybe with a lower cut-off there would have been no false negative. However, most of the literature about PEth is consistent with the 20 ng/mL as the most useful cut-off.

It is also worth looking at differences between AUDIT positive and negative patients. Not surprisingly, AUDIT positive patients had a higher rate of positive biomarkers. Actually, given the stigma that comes with alcohol dependence and the social desirability of self reports [33], one could consider biomarkers especially useful when self-reports are negative. It is also important to note that sensibility and specificity for biomarkers taking AUDIT results as the gold standard were not calculated, since this was not a validation study. While that could be seen as a limitation, it may also be considered an approach to real clinical situations where the drinking status of patients is not known beforehand. It is in that scenario where the combination of biomarkers and self-reports must be used in order to accurately evaluate patients, and in the case of liver transplantation candidates, make the appropriate decisions. Also worth emphasizing, indirect biomarkers such as transaminases and GGT values did not show any significant differences (actually were slightly higher for AUDIT negative patients).

It is also interesting to compare our study with a recently conducted study among the same population in our clinic [34]. It was a longitudinal study with several months follow-up. Alcohol screening, though, was performed only with self-reports and ethanol screening in urine specimens. Up to 76% of patients screened positive at least once during follow-up. This number is larger than the proportion we found in our study, although it must be stated that ours had a transversal, single point assessment design. It could be argued that when low sensitivity biomarkers (such as ethanol or indirect blood parameters like MCV, CDT or transaminases) are used, several months are needed to detect all cases of drinking, whereas with new direct alcohol biomarkers, a single point assessment is enough to capture the reality of this population, where not all patients are abstinent despite the importance attributed by both patients and professionals. This is indeed crucial since biomarker testing in ALD patients should not be viewed, neither by patients nor professionals, as a decision rule to exclude patients from being transplanted, but rather as a tool for early detection and treatment of drinking [27].

Head-to-head comparison between biomarkers remains difficult in this and similar studies conducted among liver transplant candidates, since these are not controlled studies where the amount of drinking ingested is known beforehand. That leads to the question whether all biomarker positive results are valid, clinical positive results. In this regard, we believe it is important to pay attention to EtS, since it was the most frequent positive test, and especially since it was the only positive biomarker in 7 patients (14% of the sample). Two previous studies testing PEth in liver transplant candidates did not include EtS testing [23,24]. Previous studies comparing EtG and EtS in the same population seem to have shown a good, even larger sensitivity for EtS compared to EtG [17]. That being said, there are also studies suggesting PEth could be more sensitive than EtS, although they have not been performed in liver transplant candidates [28].

False positive results are always an important concern, especially with alcohol-containing hand sanitizers and foods. However, with highly sensitive analysis methods such as LS/MS, the probability of such events is low [29], and it seems that EtS might be even more accurate than EtG in the distinction between ethanol ingestion and dermal exposure [35]. Another important consideration is the highly variable formation and degradation kinetics between individuals regarding EtG and EtS [30]. Moreover, some recent evidence points out toward a greater stability of EtS in human fluids when compared to EtG and PEth [36].

Worth mentioning, other authors such as Andreasen–Streichert and colleagues [23], in their liver transplant biomarkers study, considered single positive biomarkers not backed up by self-reports or any other biomarker as false positive results. While this might be a conservative approach in clinical settings, we think this might be too restrictive for research purposes.

One of the main limitations that must be stated in this and similar studies is its cross-sectional nature, where follow-up information is not gathered. As has been shown previously, this could lead to relevant information and accurate predictive values [37].

All in all, and similar to other clinical populations, a combination of biomarkers seems to be the best choice when trying to assess drinking status. Although self-reports must always be taken into account, they should always be complemented with biomarkers. The evidence gathered in this study suggests that EtS might have a significant role, as it could increase the overall sensitivity of the screening in liver transplant candidates.

## 5. Conclusions

In line with previous literature, this study suggest new direct alcohol biomarkers improve the monitorization of alcohol use disorder patients. Specifically with liver transplant candidates, direct biomarkers seem to provide more accurate information regarding drinking status, a fact of paramount importance for the proper management of such patients. 

## Figures and Tables

**Table 1:** Differences between AUDIT negative and positive patients for the main variables of the study.

Variable	AUDIT negative (score <8)	AUDIT positive (score ≥8)
Sex (% men)	83.3	87.5
Age (years)	56.8	53.6
ASAT (mean (SD))	47.6 (20) UI/L	34.6 (4) UI/L
ALAT (mean (SD))	34.9 (25) UI/L	33 (28) UI/L
GGT (mean (SD))	89.9 (86) UI/L	75.6 (11) UI/L
EtG positive (%)	2.4%	62.5%
EtS positive (%)	19.5%	62.5%
PeTH positive (%)	7.1%	25%
HEtG positive (%)	3.4%	66.7%
TLFB positive (%)	0%	37.5%
All biomarkers negative (%)	76.2%	37.5%
Other drugs used (%)	11.9%	25%
VHC (%)	28.6%	25%

AUDIT: alcohol use disorder identification test; ASAT: aspartate aminotransferase; ALAT: alanine aminotransferase; GGT: gammaglutil transferase; EtG: ethylglucuronide; EtS: ethylsulfate; PeTH: phosphatydilethanol, HEtG: hair ethylglucuronide; TLFB: timeline follow back.

**Table 2:** Main characteristics of the study biomarkers.

Biomarker	Characteristics	References
EtG	Obtained from urine. Sensible to urine infections and reduced kidney function. Suitable for abstinence monitoring. Time-frame of detection up to a few days. Cut-off 0.5 mg/L	[15,16,17,19,20,27,28,29,30]
EtS	Obtained from urine. Sensible to urine infections and reduced kidney function. Suitable for abstinence monitoring. Time-frame of detection up to a few days. Cut-off 0.05 mg/L.	[17,19,20,31,32,33]
PeTH	Obtained from dried blood spots. Suitable for abstinence and heavy drinking monitoring. Timeframe of detection from days to weeks. Cut-off 20 ng/mL	[19,21,22,23,24]
HEtG	Obtained from hair. Sensible to contamination and extraction methods. Suitable for abstinence monitoring over long periods of time. Timeframe of detection from weeks to months. Cut-off 7 pg/mL.	[19]

EtG: ethylglucuronide; EtS: ethylsulfate; PeTH: phosphatydilethanol, HEtG: hair ethylglucuronide.

## References

[1] Burra P., Senzolo M., Adam R., Delvart V., Karam V., Germani G., Neuberger J., ELITA, ELTR Liver Transplant Centers (2010). Liver Transplantation for Alcoholic Liver Disease in Europe: A Study from the ELTR (European Liver Transplant Registry). Am. J. Transpl..

[2] Burroughs A.-K., Sabin C., Rolles K., Delvart V., Karam V., Buckels J., O’Grady J.G., Castaing D., Klempnauer J., Jamieson N.V. (2006). 3-month and 12-month mortality after first liver transplant in adults in Europe: Predictive models for outcome. Lancet.

[3] Marroni N.P., Fleck A.M., Fernandes S.A., Galant L.H., Mucenic M., Meine M.H.D.M., Mariante-Neto G., Brandão A.B.D.M. (2018). Liver transplantation and alcoholic liver disease: History, controversies, and considerations. World J. Gastroenterol..

[4] Kotlyar D.S., Burke A., Campbell M.S., Weinrieb R.M. (2008). A Critical Review of Candidacy for Orthotopic Liver Transplantation in Alcoholic Liver Disease. Am. J. Gastroenterol..

[5] Testino G., Leone S. (2017). Acute alcoholic hepatitis: A literature review and proposal of treatment. Minerva Med..

[6] Frazier T.H., Stocker A.M., Kershner N.A., Marsano L.S., McClain C.J. (2010). Treatment of alcoholic liver disease. Adv. Gastroenterol..

[7] Jaurigue M.M., Cappell M.S. (2014). Therapy for alcoholic liver disease. World J. Gastroenterol..

[8] Marroni N.P. (2015). Management of alcohol recurrence before and after liver transplantation. Clin. Res. Hepatol. Gastroenterol..

[9] Dew M.A., DiMartini A.F., Steel J., Dabbs A.D.V., Myaskovsky L., Unruh M., Greenhouse J. (2008). Meta-analysis of risk for relapse to substance use after transplantation of the liver or other solid organs. Liver Transpl..

[10] Testino G. (2016). Alcohol and liver transplantation: The six-month abstinence rule is not a dogma. Transpl. Int..

[11] Testino G., Burra P., Bonino F., Piani F., Sumberaz A., Peressutti R., Castiglione A.G., Patussi V., Fanucchi T., Ancarani O. (2014). Acute alcoholic hepatitis, end stage alcoholic liver disease and liver transplantation: An Italian position statement. World J. Gastroenterol..

[12] Saunders J.B., Whitfield J.B., Conigrave K. (1995). Diagnostic tests for alcohol consumption. Alcohol Alcohol..

[13] Hock B., Schwarz M., Domke I., Grunert V.P., Wuertemberger M., Schiemann U., Horster S., Limmer C., Stecker G., Soyka M. (2005). Validity of carbohydrate-deficient transferrin (%CDT), γ-glutamyltransferase (γ-GT) and mean corpuscular erythrocyte volume (MCV) as biomarkers for chronic alcohol abuse: A study in patients with alcohol dependence and liver disorders of non-alcoholic and alcoholic origin. Addiction.

[14] Conigrave K.M., Degenhardt L.J., Whitfield J.B., Saunders J.B., Helander A., Tabakoff B., WHO/ISBRA Study Group (2002). CDT, GGT, and AST as markers of alcohol use: The WHO/ISBRA collaborative project. Alcohol. Clin. Exp. Res..

[15] Barrio P., Teixidor L., Rico N., Bruguera P., Ortega L., Bedini J.L., Gual A. (2016). Urine Ethyl Glucuronide Unraveling the Reality of Abstinence Monitoring in a Routine Outpatient Setting: A Cross-Sectional Comparison with Ethanol, Self Report and Clinical Judgment. Eur. Addict. Res..

[16] Staufer K., Andresen H., Vettorazzi E., Tobias N., Nashan B., Sterneck M. (2011). Urinary ethyl glucuronide as a novel screening tool in patients pre- and post-liver transplantation improves detection of alcohol consumption. Hepatology.

[17] Stewart S.H., Koch D.G., Burgess D.M., Willner I.R., Reuben A. (2012). Sensitivity and specificity of urinary ethyl glucuronide and ethyl sulfate in liver disease patients. Alcohol. Clin. Exp. Res..

[18] Simons J.S., Wills T.A., Emery N.N., Marks R.M. (2015). Quantifying alcohol consumption: Self-report, transdermal assessment, and prediction of dependence symptoms. Addict. Behav..

[19] Wurst F.M., Thon N., Yegles M., Schrück A., Preuss U.W., Weinmann W. (2015). Ethanol metabolites: Their role in the assessment of alcohol intake. Alcohol. Clin. Exp. Res..

[20] Allen J.P., Wurst F.M., Thon N., Litten R.Z. (2013). Assessing the drinking status of liver transplant patients with alcoholic liver disease. Liver Transpl..

[21] Stewart S.H., Reuben A., Brzezinski W.A., Koch D.G., Basile J., Randall P.K., Miller P.M. (2009). Preliminary Evaluation of Phosphatidylethanol and Alcohol Consumption in Patients with Liver Disease and Hypertension. Alcohol Alcohol..

[22] Stewart S.H., Koch D.G., Willner I.R., Anton R.F., Reuben A. (2014). Validation of Blood Phosphatidylethanol as an Alcohol Consumption Biomarker in Patients with Chronic Liver Disease. Alcohol. Clin. Exp. Res..

[23] Andresen-Streichert H., Beres Y., Weinmann W., Schröck A., Müller A., Skopp G., Pischke S., Vettorazzi E., Lohse A., Nashan B. (2017). Improved detection of alcohol consumption using the novel marker phosphatidylethanol in the transplant setting: Results of a prospective study. Transpl. Int..

[24] Fleming M.F., Smith M.J., Oslakovic E., Lucey M.R., Vue J.X., Al-Saden P., Levitsky J. (2017). Phosphatidylethanol Detects Moderate-to-Heavy Alcohol Use in Liver Transplant Recipients. Alcohol. Clin. Exp. Res..

[25] Bohn M.J., Babor T.F., Kranzler H.R. (1995). The Alcohol Use Disorders Identification Test (AUDIT): Validation of a screening instrument for use in medical settings. J. Stud. Alcohol.

[26] Sobell L.C., Sobell M.B. (1992). Timeline Follow-Back. Measuring Alcohol Consumption.

[27] Barrio P., Wurst F.M., Gual A. (2018). New Alcohol Biomarkers. New challenges. Alcohol Alcohol..

[28] Reisfield G.M., Teitelbaum S.A., Large S.O., Jones J., Morrison D.G., Lewis B. (2020). The roles of phosphatidylethanol (PEth), ethyl glucuronide (EtG), and ethyl sulfate (EtS) in identifying alcohol consumption among participants in professionals’ health programs. Drug Test. Anal..

[29] Musshoff F., Albermann E., Madea B. (2010). Ethyl glucuronide and ethyl sulfate in urine after consumption of various beverages and foods—Misleading results?. Int. J. Leg. Med..

[30] Stachel N., Skopp G. (2016). Formation and inhibition of ethyl glucuronide and ethyl sulfate. Forensic Sci. Int..

[31] Lindenger C., Castedal M., Schult A., Åberg F. (2018). Long-term survival and predictors of relapse and survival after liver transplantation for alcoholic liver disease. Scand. J. Gastroenterol..

[32] Rice J.P., Eickhoff J., Agni R., Ghufran A., Brahmbhatt R., Lucey M.R. (2013). Abusive drinking after liver transplantation is associated with allograft loss and advanced allograft fibrosis. Liver Transpl..

[33] Zemore S.E. (2012). The effect of social desirability on reported motivation, substance use severity, and treatment attendance. J. Subst. Abus. Treat..

[34] Lopez Pelayo H., Altamirano J., Lopez E., Barrio P., Lopez A., Gual A., Lligoña A. (2018). Role of Alcohol and Drug Detection by Regular Urine Sample Testing in pre-transplant evaluation for Alcohol Liver Disease. Adicciones.

[35] Reisfield G.M., Goldberger B.A., Crews B.O., Pesce A.J., Wilson G.R., Teitelbaum S.A., Bertholf R.L. (2011). Ethyl glucuronide, ethyl sulfate, and ethanol in urine after sustained exposure to an ethanol-based hand sanitizer. J. Anal. Toxicol..

[36] Liu Y., Zhang X., Li J., Huang Z., Lin Z., Wang J., Zhang C., Rao Y. (2018). Stability of Ethyl Glucuronide, Ethyl Sulfate, Phosphatidylethanols and Fatty Acid Ethyl Esters in Postmortem Human Blood. J. Anal. Toxicol..

[37] Barrio P., Mondon S., Teixidor L., Ortega L., Vieta E., Gual A. (2017). One Year Clinical Correlates of EtG Positive Urine Screening in Alcohol-Dependent Patients: A Survival Analysis. Alcohol Alcohol..

